# The Usefulness of Short‐Type Single Balloon Enteroscope for Successful Pancreato‐Biliary Cannulation During Endoscopic Retrograde Cholangiopancreatography in Patients With Roux‐en‐Y Gastrectomy: A Comparative Study With Short‐Type Double‐balloon Enteroscope

**DOI:** 10.1002/deo2.70285

**Published:** 2026-01-21

**Authors:** Hiroya Terabe, Takahiko Sakaue, Takumi Kawaguchi, Kyoyoshi Saito, Yohei Hara, Yutaka Shimamatsu, Sohei Yoshimura, Shingo Hirai, Yu Sasaki, Suketo So, Hidetoshi Takedatsu, Yoshinobu Okabe

**Affiliations:** ^1^ Division of Gastroenterology, Department of Medicine Kurume University School of Medicine Kurume Japan; ^2^ Department of Gastroenterology Tobata Kyoritsu Hospital Kitakyushu Japan; ^3^ Digestive Disease Center Kurume University Hospital Kurume Japan

**Keywords:** cannulation, retroflex position, Roux‐en‐Y gastrectomy, short‐type double‐balloon enteroscope (sDBE), short‐type single‐balloon enteroscope (sSBE)

## Abstract

**Objectives:**

The retroflex position is crucial for the success of pancreato‐biliary cannulation in patients with Roux‐en‐Y gastrectomy (RYG). We aimed to investigate the factors associated with forming the retroflex position in patients with RYG, including short‐type single‐balloon enteroscope (sSBE) and short‐type double‐balloon enteroscope (sDBE).

**Methods:**

119 consecutive patients who underwent endoscopic retrograde cholangiopancreatography (ERCP) after RYG were enrolled. All the procedures were performed using sSBE (SIF‐H290S; Olympus Medical Systems, Tokyo, Japan) or sDBE (EI‐580BT; Fujifilm, Tokyo, Japan). The clinical outcomes of ERCP were compared between patients undergoing ERCP with sSBE (*n* = 65) and sDBE (*n* = 54). A logistic regression model was used to identify the independent factors associated with retroflex position.

**Results:**

The overall cannulation success rate was 76.7% in patients with RYG. Multivariate analysis revealed that retroflex position was the only independent factor associated with successful cannulation (Odds Ratio [OR] 6.996​, 95% Confidence Interval [95%CI] 2.604–20.703, *p* = 0.0001). In the sub‐analysis using two types of scopes, sSBE, but not sDBE, was identified as an independent factor associated with the retroflex position (OR 7.025​, 95%CI 2.750–20.001, *p* = 0.0001). Decision tree analysis also revealed that the scope was the first splitting variable for the retroflex position. The retroflex position rate was 42.9% and 81.5% in patients with sDBE and sSBE, respectively.

**Conclusions:**

The retroflex position was the most useful factor for the cannulation success rate in patients with RYG. Moreover, we first demonstrated that sSBE was more useful than sDBE for forming the retroflex position. Thus, sSBE may be better for patients with RYG through easier formation of the retroflex position than sDBE.

## 1 Introduction

The endoscopic procedure is becoming commonly employed to examine or treat patients with pancreato‐biliary disease, including those who have undergone gastrointestinal surgery [1]. A major endoscopic procedure is endoscopic retrograde cholangiopancreatography (ERCP), and pancreato‐biliary cannulation is an initial step for ERCP [2]. Pancreato‐biliary cannulation is extraordinarily challenging due to several factors: the inverted papilla appearance, frequently tangential positioning, and the necessity of using a forward‐viewing endoscope without an elevator mechanism [1, 2]. Traditional endoscopic devices have consistently demonstrated low success rates in patients with Roux‐en‐Y reconstruction after gastrectomy (RYG), primarily due to the intrinsic difficulty of reaching the target site [1].

Several reports, including a multi‐center study and an expert review, demonstrate that an independent factor for the success of pancreato‐biliary cannulation is retroflex position in patients with RYG [3, 4, 5]. The retroflex position is a technique to observe the papillae in a frontal view by retroflexing the soft part of the scope. The retroflex position leads to a more than 90% cannulation success rate in patients with RYG [6]. The retroflex position is also reported to contribute to the completion of procedures such as common bile duct stone extraction after cannulation [7]. However, the retroflex position can be easily formed in not all patients. Understanding the factors that contribute to forming the retroflex position is crucial for successful pancreato‐biliary cannulation.

With the development of balloon‐assisted‐enteroscope (BE), short‐type BE (sBE) has been used in ERCP‐related procedures for pancreaticobiliary disease patients with RYG. Currently, there are two types of sBE: short‐type double‐balloon enteroscope (sDBE: EI‐580BT; Fujifilm, Tokyo, Japan) and short‐type single‐balloon enteroscope (sSBE: SIF‐H290S; Olympus Medical Systems, Tokyo, Japan). Shimatani et al. demonstrated that an sDBE has an excellent therapeutic success rate and a low rate of adverse events in the treatment of biliary disease in patients with surgically altered gastrointestinal anatomy, including RYG, in a multicenter prospective study [8]. In addition, Yane et al. demonstrated that an sSBE has a high therapeutic success rate for ERCP in postsurgical altered anatomy [9]. We also performed a multi‐center study and provided evidence that sSBE‐assisted ERCP in patients with surgically altered anatomy was effective [10]. However, these previous studies were not comparative studies for sDBE and sSBE, and it remains unclear which is more suitable for patients with RYG.

The aim of this study is to investigate independent factors for pancreato‐biliary cannulation, including retroflex position, sSBE, and sDBE in patients with RYG.

## 2 Methods

### 2.1 Study Design

This was a retrospective, two‐institution study that was approved by the Ethical Committee of Kurume University (Kurume, Japan) (Study registration no: 20222) and Tobata Kyoritsu Hospital (Kitakyushu, Japan) (Study registration no: 24‐21). We used an opt‐out approach to obtain informed consent from the patients, and personal information was protected during data collection.

### 2.2 Patients' Enrollment

We enrolled 119 consecutive patients after RYG who had undergone ERCP‐related procedures with sSBE or sDBE at Kurume University Hospital and Tobata Kyoritsu Hospital between March 2016 and October 2022. All patients had an intact papilla. We excluded patients with prior sphincterotomy, endoscopic papillary balloon dilation, or stent placement.

For analysis, patients were divided into two groups based on the endoscope used: the sSBE group (*n* = 65) and the sDBE group (*n* = 54). Patients were also classified into the success of cannulation group or the failure of cannulation group.

### 2.3 Data Collection

All data were retrospectively collected from medical records at the time of ERCP. The following information was obtained: age, sex, reason for ERCP, gastrectomy procedure, diverticulum, reaching time, total procedure time, success of reaching the papilla, success of cannulation, success of procedure, type of endoscope, the retroflex position, and adverse events.

### 2.4 ERCP Procedure and Used sSBE/sDBE

**FIGURE 1:**
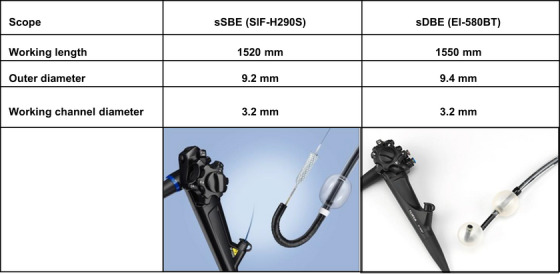
Short‐type single‐balloon enteroscope (sSBE: SIF‐H290S; Olympus Medical Systems, Tokyo, Japan) and short‐type double‐balloon enteroscope (sDBE: EI‐580BT; Fujifilm, Tokyo, Japan).

ERCP procedures at these two facilities were performed by skilled endoscopists with more than 10 years of ERCP experience under the same expert advisor. All the procedures in this study were performed using short SBE (SIF‐H290S; Olympus Medical Systems, Tokyo, Japan) or short DBE (EI‐580BT; Fujifilm, Tokyo, Japan) (Figure 1). The scope selected was the one owned by the facility. sSBE was used at Kurume University Hospital, and sDBE was used at Tobata Kyouritsu Hospital. All ERCP procedures were conducted under CO_2_ insufflation with the scope fitted with a distal attachment cap (D‐201‐10704; Olympus Medical Systems, Tokyo, Japan). The patients were basically placed in the prone position, and in cases of difficult insertion, their position was modified, or abdominal pressure was applied. If procedures were unsuccessful, reattempting ERCP, alternative approaches such as percutaneous transhepatic biliary drainage (PTBD) or endoscopic ultrasonography‐guided biliary/pancreatic drainage (EUS‐BD/PD), surgery, or conservative management were pursued.

### 2.5 Definition of Success for Pancreato‐biliary Cannulation

The success rate of biliary cannulation was defined as the rate of successful deep biliary cannulation with the obtainment of a cholangiogram in all patients with RYG who underwent ERCP as previously described. On the other hand, the failure of cannulation included patients with unreached papillae or failed cannulation.

### 2.6 Definition of Retroflex Position

**FIGURE 2:**
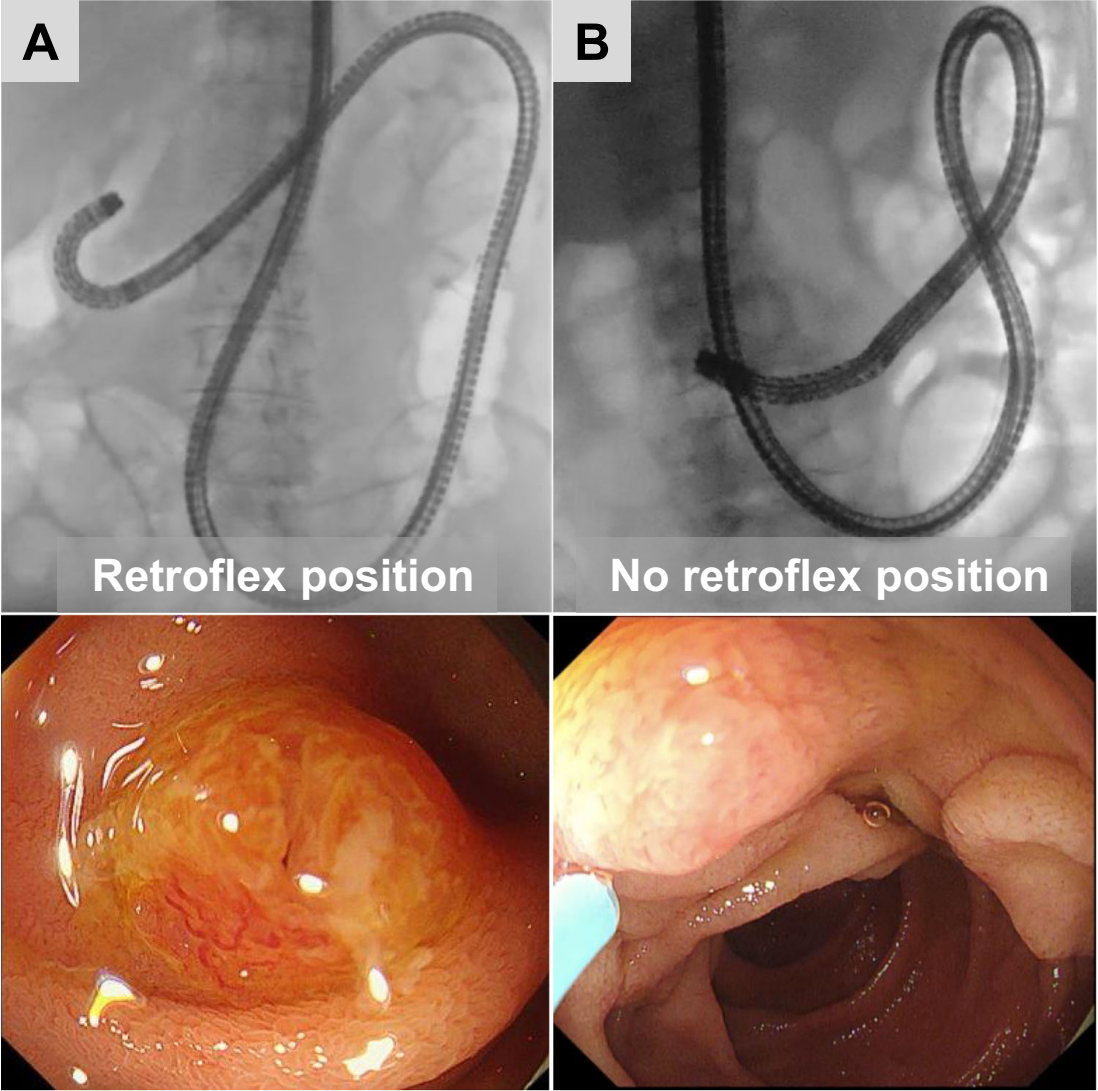
(A) Retroflex position. (B) No retroflex position. The upper panels show two radiographic images, and the lower panels show two endoscopic images.

The retroflex position was defined as the ability to observe the papilla with the scope in a J‐turn form at the inferior duodenal angle (Figure 2A). To investigate the feasibility of the retroflex position, we re‐viewed the fluoroscopic images or videos scanned during the ERCP procedure in all subjects. Figure 2B shows a case where biliary cannulation was attempted without achieving a retroflex position.

### 2.7 Statistical Analyses

Continuous variables were expressed as medians, ranges, or numbers. Categorical variables were expressed as frequencies and percentages. Differences between the groups were analyzed using the Wilcoxon rank‐sum test for continuous variables and Fisher's exact test for categorical variables. A logistic regression model was used to identify the independent factors associated with pancreato‐biliary cannulation and retroflex position. Explanatory variables were based on age, sex, reasons for ERCP, gastrectomy procedure, diverticulum, type of endoscope, retroflex position, reaching time, and total procedure time in a stepwise manner, minimizing the Bayesian information criterion, as previously described [11, 12, 13, 14]. Data were expressed as odds ratios and 95% confidence intervals; *p* < 0.05 indicated statistical significance.

The decision‐tree algorithm is a data‐mining technique that reveals a series of classification rules by identifying priorities. The explanatory variables used in the decision‐tree algorithm for pancreato‐biliary cannulation were age, sex, gastrectomy procedure, diverticulum, type of endoscope, and the retroflex position. The explanatory variables used in the decision‐tree algorithm for retroflex position were age, sex, gastrectomy procedure, diverticulum, and type of endoscope. Factors associated with pancreato‐biliary cannulation and retroflex position were automatically, but not intentionally, identified in order of importance from all explanatory variables by a statistical analysis program [15, 16, 17]. All p‐values were two‐tailed, and a value of less than 0.05 was considered statistically significant. All statistical analyses were carried out using JMP Pro17 software (SAS Institute Inc., Cary, NC).

## 3 Results

### 3.1 Comparison of Clinical Characteristics Between sSBE and sDBE Groups

The clinical characteristics of patients in the sSBE (*n* = 65) and sDBE (*n* = 54) groups are shown in Table 1. Patients in the sDBE group were significantly older than those in the sSBE group (79 vs. 75 years, *p* = 0.0197). There was a significant difference in the reasons for ERCP, with the sSBE group having a higher proportion of cases for malignant biliary obstruction (MBO) (27.7% vs. 13.0%, *p* = 0.0457). Furthermore, the type of gastrectomy differed significantly, with the sSBE group having a much higher rate of total gastrectomy (83.1% vs. 48.2%, *p* < 0.0001). There were no significant differences in sex or the presence of a diverticulum between the two groups.

**TABLE 1:** Comparison of baseline characteristics between the short‐type single‐balloon enteroscope (sSBE) and short‐type double‐balloon enteroscope (sDBE) groups.

	sSBE‐ERCP		sDBE‐ERCP		
	Percentage (number) or Median (IQR)	Range	Percentage (number) or Median (IQR)	Range	*p*‐Value
Patients (*n*)	54.6% (65/119)	N/A	45.4% (54/119)	N/A	N/A
Age (years)	75 (70‐80)	49–89	79 (72–84.3)	60–93	0.0197
Sex (Female/Male)	30.8%/69.2% (20/45)	N/A	40.7%/59.3% (22/32)	N/A	0.2575
Reasons for ERCP (CBDS or Cholangitis/MBO)	72.3%/27.7% (47/18)	N/A	87.0%/13.0% (47/7)	N/A	0.0457
Gastrectomy procedure (Total gastrectomy with RY/Partial gastrectomy with RY)	83.1%/16.9 (54/11)	N/A	48.2%/51.9%	N/A	<0.0001
Diverticulum (Presence/Absence)	14.0%/86.0% (8/49)	N/A	18.5%/81.5% (10/44)	N/A	0.5217

*Note*: Data are expressed as median (interquartile range [IQR]), range, or number.

Abbreviations: CBDS, common bile duct stones; ERCP, endoscopic retrograde cholangiopancreatography; MBO, malignant biliary obstruction; N/A, not applicable; RY, Roux‐en‐Y; sDBE, short‐type double‐balloon enteroscope; sSBE, short‐type single balloon enteroscope.

### 3.2 Procedural Outcomes and Adverse Events

**FIGURE 3:**
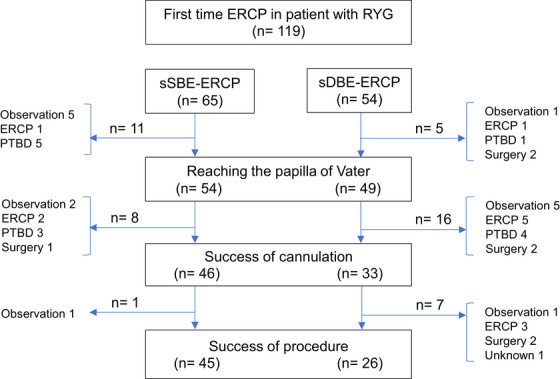
Flowchart of the procedural outcomes. The diagram shows the clinical course of all 119 patients. Patients were divided into two groups based on the endoscope used (short‐type single‐balloon enteroscope [sSBE] or short‐type double‐balloon enteroscope [sDBE]). The chart details the number of successful and unsuccessful cases at each procedural step: reaching the papilla of Vater, successful cannulation, and final procedural success. The subsequent management for patients with procedural failure is also summarized.

Figure 3 provides a flowchart of the procedural outcomes. A comparison of the outcomes between the sSBE and sDBE groups is detailed in Table 2.

**TABLE 2:** Comparison of procedural outcomes between the short‐type single‐balloon enteroscope (sSBE) and short‐type double‐balloon enteroscope (sDBE) groups.

	sSBE‐ERCP		sDBE‐ERCP		
	Percentage (number) or Median (IQR)	Range	Percentage (number) or Median (IQR)	Range	*p*‐Value
Reaching the papilla of Vater	83.1% (54/65)	N/A	90.7% (49/54)	N/A	0.2160
Success of cannulation	85.2% (46/54)	N/A	67.4% (33/49)	N/A	0.0315
Success of the procedure	97.8% (45/46)	N/A	78.8% (26/33)	N/A	0.0045
Retroflex position (Yes)	81.5% (44/54)	N/A	42.9% (21/49)	N/A	<0.0001
Reaching time (min)	24 (13.8–42.3)	7–150	15 (11–43)	4‐70	0.0054
Cannulation time (min)	11 (5–24.3)	1–88	15 (7–28.5)	2‐73	0.3340
Total procedure time (min)	92 (70–120)	23–240	84 (63–110)	32‐131	0.0371

*Note*: Data are expressed as median (interquartile range [IQR]), range, or number.

Abbreviations: ERCP, endoscopic retrograde cholangiopancreatography; N/A, not applicable; sDBE, short‐type double‐balloon enteroscope; sSBE, short‐type single balloon enteroscope.

There was no significant difference in the success rate of reaching the papilla of Vater. However, statistically significant differences were observed between the two groups in the overall cannulation success rate (85.2% vs. 67.4%, *p* = 0.0315) and the overall procedure success rate (97.8% vs. 78.8%, *p* = 0.0045). The achievement of the retroflex position was significantly more frequent in the sSBE group (81.5% vs. 42.9%, *p* < 0.0001). No significant difference was observed in bile duct cannulation time; however, the median reaching time and total procedure time were significantly longer in the sSBE group compared to the sDBE group. As detailed in Table 3, adverse events occurred in 20 patients (16.8%), with the sSBE group significantly more frequent than the sDBE group.

**TABLE 3:** Adverse events.

	sSBE‐ERCP		sDBE‐ERCP		
	Percentage (number) or Median (IQR)	: Severity	Percentage (number) or Median (IQR)	: Severity	*p*‐Value
**Adverse events**	23.0% (15/65)		9.3% (5/54)		0.0448
Pancreatitis	9.2% (6): mild 4, moderate 2		3.7% (2): mild 1, moderate 1		
Hyperamylasemia	9.2% (6): mild 6		1.9% (1): Mild 1		
Cholangitis	0% (0)		1.9% (1): Mild 1		
Perforation	1.5% (1): severe 1		1.9% (1): severe 1		
Transient hypotension	1.5% (1): mild 1		0% (0)		

*Note*: Data are expressed as median (interquartile range [IQR]), range, or number.

Abbreviations: ERCP, endoscopic retrograde cholangiopancreatography; sDBE, short‐type double‐balloon enteroscope; sSBE, short‐type single balloon enteroscope.

### 3.3 Reasons for Cannulation Failure

We investigated the reasons for cannulation failure among the 24 patients in whom cannulation was unsuccessful despite reaching the papilla. In the sSBE group (*n* = 8), the reasons for failure were intradiverticular papilla (*n* = 2), invasion of malignant tumor (*n* = 2), difficulty with axis alignment or frontal view of papilla (*n* = 1), intestinal adhesion (*n* = 1), and complication of intestinal perforation (*n* = 1). In the sDBE group (*n* = 16), the reasons for failure were difficulty with axis alignment or frontal view of papilla (*n* = 11), intradiverticular papilla (*n* = 4), and intestinal adhesion (*n* = 1).

### 3.4 Comparison of Clinical Characteristics Between Cannulation Success and Failure Groups

The clinical characteristics of patients in the cannulation success and failure groups are shown in Table 4. Among 103 patients who reached the papilla of Vater, the overall cannulation success rate was 76.7% (79/103).

**TABLE 4:** Comparison of characteristics between the cannulation success and failure groups.

	Success of cannulation		Failure of cannulation		
	Percentage (number) or Median (IQR)	Range	Percentage (number) or Median (IQR)	Range	*p*‐Value
Patients (*n*)	76.7% (79/103)	N/A	23.3% (24/103)	N/A	N/A
Age (years)	76 (70–82)	56–89	79 (72–85)	54–93	0.2864
Sex (Female/Male)	29.1%/70.9% (23/56)	N/A	50.0%/50.0% (12/12)	N/A	0.0631
Reasons for ERCP (CBDS+ Cholangitis/MBO)	86.1%/13.9% (68/11)	N/A	75.0%/25.0% (18/6)	N/A	0.2178
Gastrectomy procedure (Total gastrectomy with RY/Partial gastrectomy with RY)	67.1%/32.9% (53/26)	N/A	62.5%/37.5% (15/9)	N/A	0.6793
Diverticulum (Presence/Absence)	15.2%/84.8% (12/67)	N/A	27.3%/72.7% (6/16)	N/A	0.2080
Type of endoscope (sSBE/sDBE)	58.2%/ 41.7% (46/33)	N/A	33.3%/66.7% (8/16)	N/A	0.0315
Reaching the papilla of Vater	76.7% (79/103)	N/A	23.3% (24/103)	N/A	N/A
Success of the procedure	89.9% (71/79)	N/A	10.1% (8/79)	N/A	N/A
Retroflex position (Yes/No)	73.4%/26.6% (58/21)	N/A	29.2%/70.8% (7/17)	N/A	<0.0001
Reaching time (min)	17 (13‐38)	4‐150	18.5 (11.3‐42.3)	7‐90	0.6395
Total procedure time (min)	95 (70‐111)	23‐240	82 (58‐118)	32‐140	0.1485

*Note*: Data are expressed as median (interquartile range [IQR]), range, or number.

Abbreviations: CBDS, common bile duct stones; ERCP, endoscopic retrograde cholangiopancreatography; MBO, malignant biliary obstruction; N/A, not applicable; RY, Roux‐en‐Y; sDBE, short‐type double‐balloon enteroscope; sSBE, short‐type single balloon enteroscope.

There was no significant difference in age, sex, the reason for ERCP, the type of gastrectomy, and the presence of diverticulum between the success and failure groups. In contrast, the type of endoscope used was significantly different, with sSBE being used more frequently in the success group (58.2% vs. 33.3%, *p* = 0.0315). Moreover, the retroflex position was achieved in a significantly higher proportion of patients in the success group (73.4% vs. 29.2%, *p* < 0.0001).

### 3.5 Independent Factors for Successful Cannulation and Retroflex Position (Multivariate Analysis)

To identify the independent factors for successful cannulation, a multivariate analysis was performed on the 103 patients in whom the scope successfully reached the papilla of Vater. The analysis revealed that the retroflex position was the only independent factor significantly associated with successful cannulation (Odds Ratio [OR] 6.996, 95% Confidence Interval [CI] 2.604–20.703, *p* = 0.0001) (Table 5).

**TABLE 5:** Independent factors associated with pancreato‐biliary cannulation in patients with Roux‐en‐Y gastrectomy (RYG).

Factor	Odds ratio	95% CI	*p*‐Value
Retroflex position (Yes)	6.996	2.604–20.703	0.0001

Abbreviations: CI, confidence interval; RYG, gastrectomy with Roux‐en Y.

Subsequently, we performed a multivariate analysis to identify independent factors associated with achieving the retroflex position (Table 6). This analysis identified the type of endoscope (sSBE) (OR 7.025, 95%CI 2.750–20.001, *p* = 0.0001) and the Reasons for ERCP (CBDS + Cholangitis) (OR 4.062, 95%CI 1.196–14.996, *p* = 0.0245) as significant independent factors.

**TABLE 6:** Independent factors associated with retroflex position.

Factors	Odds ratio	95% CI	*p*‐Value
Scope (sSBE)	7.025	2.750‐20.001	0.0001
Reasons for ERCP (CBDS + Cholangitis)	4.062	1.196–14.996	0.0245

Abbreviations: CBDS, common bile duct stones; CI, confidence interval; ERCP, endoscopic retrograde cholangiopancreatography; sSBE, short‐type single balloon enteroscope.

### 3.6 Decision‐Tree Analysis for Profiles Associated With Pancreato‐Biliary Cannulation

**FIGURE 4:**
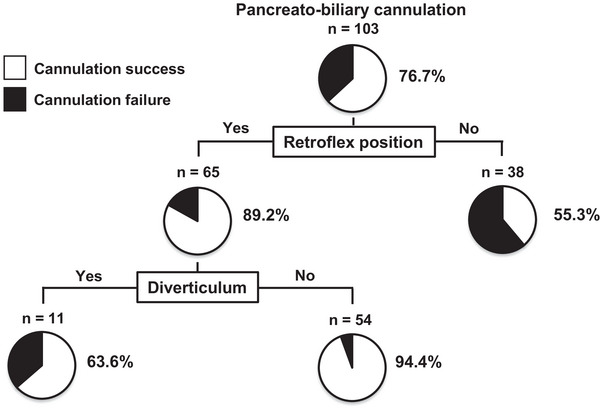
Decision‐tree analysis for profiles associated with pancreato‐biliary cannulation. The pie graphs indicate the proportions of patients with cannulation success (white) and patients with cannulation failure (black).

We performed a decision tree analysis to determine the profiles associated with successful pancreato‐biliary cannulation (Figure 4). The retroflex position was the first splitting variable for successful cannulation. The successful cannulation rate was 55.3% in patients with the absence of the retroflex position. In contrast, the rate was 89.2% in patients with the presence of the retroflex position. Of patients with the presence of the retroflex position, the second splitting variable was diverticulum. The successful cannulation rates were 94.4% in patients with the absence of the diverticulum (Figure 4).

### 3.7 Decision‐Tree Analysis for Profiles Associated With the Retroflex Position

**FIGURE 5:**
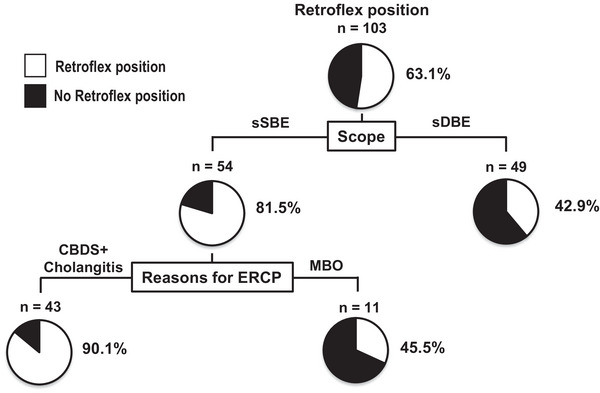
Decision‐tree analysis for profiles associated with the retroflex position. The pie graphs indicate the proportions of patients with the retroflex position (white) and patients without the retroflex position (black).

We performed a decision tree analysis to determine the profiles associated with the retroflex position (Figure 5). The scope was the first splitting variable for the retroflex position. The retroflex position rate was 39% in patients with sDBE. In contrast, the rate was 67% in patients with sSBE (Profile 1 in Figure 5). Of patients with sSBE, the second splitting variable was the reason for ERCP. The retroflex position rates were 90.1% in patients with CBDS (Figure 5).

## 4 Discussion

This study investigated factors associated with pancreato‐biliary cannulation to the naive papilla during the ERCP procedure in patients with RYG. We demonstrated that the success rate for the cannulation was 76.7%. Moreover, we found that the retroflex position was the only independent factor and the first splitting variable for successful cannulation. Furthermore, a decision‐tree analysis revealed that sSBE, but not sDBE, was the most distinguished factor associated with the retroflex position.

We demonstrated that the success rate for the cannulation of naive papilla was 76.7% in patients with RYG. Itoi et al. reported a 66.7% success rate for bile duct cannulation in patients with RY anastomosis and an intact papilla using short‐type or long‐type DBE [18]. Similarly, several reports using SBE have stated that the success rate of bile duct cannulation was 70%–74% [19, 20, 21]. Accordingly, our results were in good agreement with the results of previous reports, suggesting that our endoscopic techniques were considered to be of a comparable standard.

We found that the retroflex position was the only independent factor and the first splitting variable for successful cannulation, with an 89.2% success rate. Tanisaka et al. reported that the retroflex position is a useful technique for cannulation using sSBE [3]. In addition, Ishii et al. reported that the retroflex position is a useful technique used to obtain a better view of the papilla in RYG cases using SBE or DBE [22]. A possible reason is that the retroflex position can be a position with a coaxial relationship between the devices and the distal CBD. In addition, another possible reason is that the retroflex position can maintain a proper distance from the tip of the scope to the papilla of Vater with a better view of the papilla [3]. Our results, along with previous reports, indicated that the retroflex position was the most important factor for cannulation in patients with RYG.

There are no reports directly comparing the effectiveness of SBE versus DBE for taking the retroflex position in ERCP for patients after RY reconstruction. In our study, we revealed that sSBE, but not sDBE, was the most distinguished factor associated with the retroflex position. Although it is not clear why SBE was more useful than DBE in this study, two possible explanations can be considered: the interaction between the differences in the tip of the SBE and the interaction with anatomical features. First, SBE facilitates easier J‐turns than DBE because it does not have a balloon at the tip [23], requires a rubber to hold the balloon in place [24], and possesses passive bending functionality. The reason for the superiority of the SBE is thought to be that the tip of the SBE has a structure that is more suitable for forming the retroflex position than that of the DBE. Second, the differing mechanical properties of each endoscope may interact with anatomical variations such as the length and mobility of the afferent limb or the presence of adhesions.

There are several limitations in our study. First, this was a retrospective study conducted at two different institutions. Specifically, the use of sSBE at a university hospital and sDBE at a community hospital introduces a significant potential for selection bias, as patient populations, referral patterns, and operator experience may inherently differ. Second, as shown in Table 1, there were significant differences in baseline patient characteristics, such as age, reason for ERCP, and type of gastrectomy, between the two groups. Although we performed a multivariable analysis to adjust for these confounding factors, the possibility of residual bias cannot be eliminated. Third, the achievement rate of the retroflex position was significantly higher in the SBE group. While we suggest this may be due to the structural advantages of the sSBE scope tip, we cannot rule out the influence of different procedural policies or unmeasured technical preferences between the two institutions. This point should be considered a key limitation when interpreting the superiority of sSBE in forming a retroflex position. Finally, the number of enrolled patients was small. Despite these limitations, the advantage of this study is that it is the first to directly compare procedural outcomes between sSBE and sDBE in this challenging patient population. Our findings should be validated by a future prospective, multi‐center study.

In summary, in patients with Roux‐en‐Y gastrectomy, the retroflex position was the most useful factor for the cannulation success rate. Furthermore, we first demonstrated that SBE was more useful than DBE for forming the retroflex position. Thus, SBE was considered a useful scope for cannulation of RYG patients through an easier forming retroflex position compared to the DBE.

## Authors Contributions

H.T., T.S., T.K., and Y.O. substantially contributed to the study's conceptualization and manuscript drafting. K.S., Y.H., Y.S., S.Y., S.H., and Y.S. significantly contributed to data analysis and interpretation. S.S. and H.T. supervised the conduct of this study. All authors critically reviewed and revised the manuscript draft and approved the final version for submission.

## Conflicts of Interest

Takumi Kawaguchi received lecture fees from Nippon Boehringer Ingelheim Co., Ltd., Sumitomo Pharma Co., Ltd., Novo Nordisk Pharma Ltd., Otsuka Pharmaceutical Co., Ltd., and Janssen Pharmaceutical K.K. Yoshinobu Okabe received lecture fees from Kaneka Medix Co., Ltd., SB‐KAWASUMI Co., Ltd., and Gadelius Medical K.K. The other authors declare no conflicts of interest.

## Funding

The authors received no specific funding for this work.

## Ethics Statement

This study was conducted in accordance with the Declaration of Helsinki. Ethical approval was obtained from the Ethical Committee of Kurume University (Kurume, Japan) (Study registration no: 20222) and Tobata Kyoritsu Hospital (Kitakyushu, Japan) (Study registration no: 24‐21).

## Consent

Informed consent was obtained from all patients using an opt‐out approach. This article does not contain any studies with animal subjects performed by any of the authors.

## References

[1] A. Forbes and P. B. Cotton , “ERCP and Sphincterotomy After Billroth II Gastrectomy,” Gut 25 (1984): 971–974. 6469083 10.1136/gut.25.9.971 PMC1432487

[2] R. E. Hintze , A. Adler , W. Veltzke , et al., “Endoscopic Access to the Papilla of Vater for Endoscopic Retrograde Cholangiopancreatography in Patients With Billroth II or Roux‐en‐Y Gastrojejunostomy,” Endoscopy 29 (1997): 69–73. 9101141 10.1055/s-2007-1004077

[3] Y. Tanisaka , S. Ryozawa , M. Mizuide , et al., “Biliary Cannulation in Patients With Roux‐en‐Y Gastrectomy: An Analysis of the Factors Associated With Successful Cannulation,” Internal Medicine 59 (2020): 1687–1693. 32296000 10.2169/internalmedicine.4245-19 PMC7434537

[4] T. Obata , K. Tsutsumi , H. Kato , et al., “Balloon Enteroscopy‐Assisted Endoscopic Retrograde Cholangiopancreatography for the Treatment of Common Bile Duct Stones in Patients With Roux‐en‐Y Gastrectomy: Outcomes and Factors Affecting Complete Stone Extraction,” Journal of Clinical Medicine 10 (2021): 3314. 34362098 10.3390/jcm10153314 PMC8348346

[5] M. Shimatani , T. Mitsuyama , T. Yamashina , et al., “Advanced Technical Tips and Recent Insights in ERCP Using Balloon‐assisted Endoscopy,” DEN Open 4 (2024): e301. 38023665 10.1002/deo2.301 PMC10644950

[6] M. J. Yang , J. H. Kim , J. C. Hwang , et al., “Mechanistic Loop Resolution Strategy for Short‐type Single‐balloon Enteroscopy‐assisted Endoscopic Retrograde Cholangiopancreatography in Patients With Roux‐en‐Y Reconstruction After Gastrectomy (With video),” Surgical Endoscopy 36 (2022): 8690–8696. 36136178 10.1007/s00464-022-09575-2

[7] R. A. Cridland and N. W. Kasting , “A Simple Cannula Assembly for Chronic Bilateral Brain Infusion in Freely Moving Rats,” Brain Research Bulletin 32 (1993): 201–204. 8348346 10.1016/0361-9230(93)90076-n

[8] M. Shimatani , H. Hatanaka , H. Kogure , et al., “Diagnostic and Therapeutic Endoscopic Retrograde Cholangiography Using a Short‐Type Double‐Balloon Endoscope in Patients with Altered Gastrointestinal Anatomy: A Multicenter Prospective Study in Japan,” American Journal of Gastroenterology 111 (2016): 1750–1758. 27670601 10.1038/ajg.2016.420

[9] K. Yane , A. Katanuma , H. Maguchi , et al., “Short‐type Single‐balloon Enteroscope‐assisted ERCP in Postsurgical Altered Anatomy: Potential Factors Affecting Procedural Failure,” Endoscopy 49 (2017): 69–74. 27760436 10.1055/s-0042-118301

[10] Y. Tanisaka , S. Ryozawa , T. Itoi , et al., “Efficacy and Factors Affecting Procedure Results of Short‐type Single‐balloon Enteroscopy‐assisted ERCP for Altered Anatomy: A Multicenter Cohort in Japan,” Gastrointestinal Endoscopy 95 (2022): 310–318. 34534494 10.1016/j.gie.2021.09.008

[11] M. Nakano , M. Kawaguchi , T. Kawaguchi , et al., “Profiles Associated With Significant Hepatic Fibrosis Consisting of Alanine Aminotransferase >30 U/L, Exercise Habits, and Metabolic Dysfunction‐Associated Steatotic Liver Disease,” Hepatology Research 54 (2024): 655–666. 38294999 10.1111/hepr.14020

[12] T. Sano , K. Amano , T. Ide , et al., “Metabolic Management After Sustained Virologic Response in Elderly Patients With Hepatitis C Virus: A Multicenter Study,” Hepatology Research 54 (2024): 326–335. 37975277 10.1111/hepr.13993

[13] S. Shimose , R. Sugimoto , A. Hiraoka , et al., “Significance of Ramucirumab Following Atezolizumab plus Bevacizumab Therapy for Hepatocellular Carcinoma Using Real‐world Data,” Hepatology Research 53 (2023): 116–126. 36316794 10.1111/hepr.13852

[14] T. Sano , K. Amano , T. Ide , et al., “A Combination of Hepatic Encephalopathy and Body Mass Index Was Associated With the Point of No Return for Improving Liver Functional Reserve After Sofosbuvir/Velpatasvir Treatment in Patients With hepatitis C Virus‐related Decompensated Cirrhosis,” Hepatology Research 53 (2023): 26–34. 36066400 10.1111/hepr.13837

[15] T. Nakane , S. Fukunaga , D. Nakano , et al., “Impact of Metabolic Dysfunction‐associated Fatty Liver Disease on the Incidence of Helicobacter pylori‐negative Gastric Cancer,” Hepatology Research 54 (2024): 540–550. 38156966 10.1111/hepr.14010

[16] S. Fukunaga , M. Mukasa , T. Nakane , et al., “Impact of Non‐obese Metabolic Dysfunction‐associated Fatty Liver Disease on Risk Factors for the Recurrence of Esophageal Squamous Cell Carcinoma Treated With Endoscopic Submucosal Dissection: A Multicenter Study,” Hepatology Research 54 (2024): 201–212. 37796562 10.1111/hepr.13973

[17] S. Shimose , A. Hiraoka , A. Casadei‐Gardini , et al., “The Beneficial Impact of Metabolic Dysfunction‐Associated Fatty Liver Disease on Lenvatinib Treatment in Patients With Non‐viral Hepatocellular Carcinoma,” Hepatology Research 53 (2023): 104–115. 36149726 10.1111/hepr.13843

[18] T. Itoi , K. Ishii , A. Sofuni , et al., “Long‐ and Short‐type Double‐balloon Enteroscopy‐Assisted Therapeutic ERCP for Intact Papilla in Patients With a Roux‐en‐Y Anastomosis,” Surgical Endoscopy 25 (2011): 713–721. 20976503 10.1007/s00464-010-1226-4 PMC3044838

[19] A. Saleem , T. H. Baron , C. J. Gostout , et al., “Endoscopic Retrograde Cholangiopancreatography Using a Single‐balloon Enteroscope in Patients With Altered Roux‐en‐Y Anatomy,” Endoscopy 42 (2010): 656–660. 20589594 10.1055/s-0030-1255557

[20] T. Itoi , K. Ishii , A. Sofuni , et al., “Single‐balloon Enteroscopy‐Assisted ERCP in Patients With Billroth II Gastrectomy or Roux‐en‐Y Anastomosis (With video),” American Journal of Gastroenterology 105 (2010): 93–99. 19809409 10.1038/ajg.2009.559

[21] T. Kawamura , K. Uno , A. Suzuki , et al., “Clinical Usefulness of a Short‐Type, Prototype Single‐Balloon Enteroscope for Endoscopic Retrograde Cholangiopancreatography in Patients With Altered Gastrointestinal Anatomy: Preliminary Experiences,” Digestive Endoscopy 27 (2015): 82–86. 25040667 10.1111/den.12322

[22] K. Ishii , T. Itoi , R. Tonozuka , et al., “Balloon Enteroscopy‐Assisted ERCP in Patients With Roux‐en‐Y Gastrectomy and Intact Papillae (With videos),” Gastrointestinal Endoscopy 83 (2016): 377–386.e6. 26234697 10.1016/j.gie.2015.06.020

[23] J. T. E. Koh , L. Kim Wei , and C. P. Francisco , “Double Balloon Enteroscopy Versus Single Balloon Enteroscopy: A Comparative Study,” Medicine 103 (2024): e38119. 38758917 10.1097/MD.0000000000038119 PMC11098199

[24] M. Manno , C. Barbera , H. Bertani , et al., “Single Balloon Enteroscopy: Technical Aspects and Clinical Applications,” World Journal of Gastroenterology 4 (2012): 28–32. 10.4253/wjge.v4.i2.28 PMC3280352 22347529

